# Impact of Bep or Carboplatin Chemotherapy on Testicular Function and Sperm Nucleus of Subjects with Testicular Germ Cell Tumor

**DOI:** 10.3389/fphar.2016.00122

**Published:** 2016-05-13

**Authors:** Marco Ghezzi, Massimiliano Berretta, Alberto Bottacin, Pierfrancesco Palego, Barbara Sartini, Ilaria Cosci, Livio Finos, Riccardo Selice, Carlo Foresta, Andrea Garolla

**Affiliations:** ^1^Unit of Andrology and Reproductive Medicine, Department of Medicine, University of PadovaPadova, Italy; ^2^Istituto Oncologico Veneto – Istituto di Ricovero e Cura a Carattere ScientificoPadova, Italy; ^3^Department of Medical Oncology, CRO Aviano National Cancer Institute IRCCSAviano, Italy; ^4^Department of Statistical Sciences, University of PadovaPadova, Italy

**Keywords:** BEP, carboplatin, chemotherapy, fertility, testis cancer

## Abstract

Young males have testicular germ cells tumors (TGCT) as the most common malignancy and its incidence is increasing in several countries. Besides unilateral orchiectomy (UO), the treatment of TGCT may include surveillance, radiotherapy, or chemotherapy (CT), basing on tumor histology and stage of disease. It is well known that both radio and CT may have negative effects on testicular function, affecting spermatogenesis, and sex hormones. Many reports investigated these aspects in patients treated with bleomycin, etoposide, and cisplatin (BEP), after UO. In contrast no data are available on the side effects of carboplatin treatment in these patients. We included in this study 212 consecutive subjects who undergone to sperm banking at our Andrology and Human Reproduction Unit after UO for TGCT. Hundred subjects were further treated with one or more BEP cycles (BEP-group), 54 with carboplatin (CARB group), and 58 were just surveilled (*S*-group). All patients were evaluated for seminal parameters, sperm aneuploidy, sperm DNA, sex hormones, volume of the residual testis at baseline (T0) and after 12 (T1) and 24 months (T2) from UO or end of CT. Seminal parameters, sperm aneuploidies, DNA status, gonadic hormones, and testicular volume at baseline were not different between groups. At T1, we observed a significant reduction of sperm concentration and sperm count in the BEP group versus baseline and versus both Carb and *S*-group. A significant increase of sperm aneuploidies was present at T1 in the BEP group. Similarly, the same group at 1 had altered sperm DNA integrity and fragmentation compared with baseline, *S*-group and Carb group. These alterations were persistent after 2 years from the end of BEP treatment. Despite a slight improvement at T2, the BEP group had still higher percentages of sperm aneuploidies than other groups. No impairment of sperm aneuploidies and DNA status were observed in the Carb group both after 1 and 2 years from the end of treatment. Despite preliminary, these data demonstrate that in selected patients with TGCTs CT with carboplatin represents a therapeutic option that that seems to not affect sex hormones, spermatogenesis, and sperm nucleus.

## Introduction

Young males aged 15–40 years have testicular germ cells tumors (TGCTs) as the most common malignancy that may affect their health status (Schmoll et al., 2010; Oldenburg et al., 2013).

It has been reported that about 18,000 European subjects over reproductive age develop a TGCTs every year (Znaor et al., 2014) and its incidence is increasing in several countries over the past 50 years (Huyghe et al., 2007). Many risk factors have been studied as a pre-disposing factor in the development of this cancer, but only for some there is a high level of evidence (Garolla et al., 2012; Ferlin and Foresta, 2014). Early diagnosis and modern treatment have resulted in over 95% survival rate and improved quality of life of testicular cancer survivors (Schagen et al., 2008; Ping et al., 2014).

Usually, treatment for TGCTs is a combination of orchiectomy and radiation therapy (RT), platinum-based chemotherapy (CT) or just surveillance, based on tumor histology and stage of disease. CT including alkylating agents or RT directed to the gonads are usually used for stage II and relapsed diseases. These treatments have well-known negative side effects on testicular functions affecting both spermatogenesis and steroidogenesis up to oligo/azoospermia and hypogonadism. Moreover a negative impact of cancer treatments on sperm DNA and chromosomes has been shown after years from the end of therapy (Tempest et al., 2008; O’Flaherty et al., 2010; Patassini et al., 2013). It was proposed that, between CT drugs, etoposide appears to be a good candidate for aneuploidy sperm induction as it interacts with topoisomerase II during anaphase I and II of meiosis (De Mas et al., 2001). Besides the alteration of spermatogenesis, bleomycin, etoposide and cisplatin (BEP)-use has been reported to affect sperm chromosome and DNA status (De Mas et al., 2001). These authors, investigating sperm cell aneuploidy for chromosomes 7, 16, 18, X, and Y in a cohort of BEP-treated TGCTs described a significant increase in the frequency of diploidy and disomy for these chromosomes compared to healthy controls. In a subsequent study done in a population of TGCTs patients, Tempest et al. (2008) documented an increase of aneuploidies for sperm chromosomes, X, Y, 13, and 21, 6 months after the end of 2–4 BEP cycles that decline to pretreatment levels only at 18 months after the end of treatment. Based on these findings, some authors suggested that treated patients should be counseled to avoid conceptions up to 2 years after the end of treatments (Hassold et al., 1996; Tempest et al., 2008).

Exposure to the treatment regimen for testis cancer (BEP or cyclophosphamide) causes DNA damage, inducing cross-links as well as single and double strand breaks: therefore these treatments may represent a potential risk to subsequent generations if DNA and telomere damage in sperm are sustained; moreover it was reported that significant sperm DNA damage and reduced chromatin compaction persisted in men for up to 24 months after BEP treatment (Zhou and Bartek, 2004; O’Flaherty et al., 2010; Liu et al., 2015). Recently Liu et al. (2015) demonstrated that *in vivo* exposure of rat male germ cells to a BEP regimens, analogous to that given to testis cancer patients, induces telomere shortening in all stages of spermatogenesis raising concern about the potential risk of BEP exposure to subsequent generations. Interestingly DNA double strand breaks were increased significantly in zygotene rat male germ cells exposed to BEP regimens, as assessed by gamma-H2AX immunofluorescence (Liu et al., 2015). Furthermore a marked increase in DNA Fragmentation Index after 3 and 6 months to the end of BEP treatment was demonstrated in a series of TGCTs patients, improving after 12 and 24 months to the end of treatments (Paoli et al., 2015).

On this basis, patients candidate to CT usually receive medical counseling about sperm preservation, endocrine follow-up and genetic counseling to avoid conceptions during the treatments period up to 2 years after the end of therapy aimed to avoid possible defective genome transmission to their offspring (Tempest et al., 2008; Ping et al., 2014). Despite some authors raised concern about the hypothesis that spermatozoa may carry damages DNA even long after treatments have finished, there is no actual evidence to suggest that previous TGCTs treatments are associated with birth defect (Thomson et al., 2002; Ping et al., 2014).

Because most patients and their parents are interested to preserve fertility, sperm banking should be recommended before cancer treatments (Jeruss and Woodruff, 2009). Although sperm cryopreservation allow cancer survivors to reach fertility with *in vitro* fertilization (IVF), new treatment strategies with a lower impact on testicular function are challenging to preserve spontaneous fertility and to reduce sperm DNA alterations and hormones impairments avoiding potential hypogonadism (Choy and Brannigan, 2013). Many reports have investigated the impact on testicular function and in particular on spermatogenesis of RT and CT with BEP in men before and after orchiectomy (Thomson et al., 2002; Bujan et al., 2013; Choy and Brannigan, 2013). In contrast no data are available on the effects of a carboplatin (CARB)-based CT on these topics and, in particular, no data are available on the comparison between BEP-treated and CARB-treated subjects.

Aim of the present study was to assess the impact of orchiectomy followed by CT (BEP treatment, or surveillance alone on sperm parameters, sperm aneuploidies and DNA fragmentation, sex hormones, and testicular volume in TGCTs cancer patients at basal and at 12 and 24 months after the end of any treatment. In particular, we aimed to evaluate the possible gonadotoxic effects of CARB in comparison with surveillance and BEP treatments.

## Materials and Methods

### Subjects

This retrospective study enrolled 212 consecutively subjects who had been sent by oncologist to Unit of Andrology and Reproductive Medicine of Padua for sperm banking after unilateral orchiectomy (UO) for TGCTs. Patients had a cancer stage ranging from first to third. The subjects were divided into three groups based on the management of disease suggested by the oncologist: 100 with one to four BEP cycles based on tumor histology, 54 with CARB one cycle (group 3), and 58 had no treatment (surveillance alone). Patients were evaluated at baseline (T0) and then followed-up after 12 and 24 months from the start of treatments (T1 and T2, respectively) for sperm parameters, sex hormones, testicular volume, sperm aneuploidies, and DNA integrity. We have excluded from the study patients with know causes of infertility such as Klinefelter syndrome, Y chromosome microdeletions, varicocele in the remaining testis and history of orchitis or cryptorchidism. Written informed consent was obtained from all patients, and the study protocol was approved by the local ethics committee.

### Semen Sample Collection and Processing

Semen samples were obtained by masturbation after 2–5 days of sexual abstinence. After liquefaction at room temperature, semen volume, pH, sperm concentration, total sperm count (TSC), viability, motility, and normal morphology were determined according to World Health Organization guidelines for semen analysis (World Health Organization [WHO], 1999). Semen samples were then washed three times with sterile phosphate-buffered saline (PBS 1), and the pellet was used for the subsequent analyses.

### Hormone Assay

Blood sample was collected in the fasting state between 08.00 and 1000 h. Serum FSH, LH, and total testosterone were evaluated by commercial electrochemiluminescence immunoassay methods (Elecsys, 2010; Roche Diagnostics, Mannheim, Germany) as reported elsewhere (Selice et al., 2011). For all parameters, the intra- and inter-assay coefficients of variation were <8 and 10%, respectively).

### Ultrasound (US) Scanning

To evaluate testicular size, morphology and normal tissue echo pattern, all subjects were studied with ultrasonography. All US examinations were made using a high resolution machine (Aplio XV Toshiba, Tokyo, Japan) equipped with a 7–13 MHz multifrequency linear probe by the same experienced doctor (P.P.). Intra-observer variability for testicular volume was estimated to be less than 10% as reported in a previous study of our group (Selice et al., 2011).

### FISH Analysis for Sperm Aneuploidy

The study of sperm aneuploidy was performed by multicolor FISH, as reported elsewhere (Garolla et al., 2013). The DNA hybridization was performed using a human satellite probe-specific mix for chromosomes X, Y, and 18 (Kreatech Diagnostics). Probes were direct-labeled with fluorochrome PlatinumBright495 for chromosome X, resulting in a green signal and fluorochrome PlatinumBright550 for chromosome Y, resulting in a red signal, for the detection of chromosome 18 a PlatinumBright415 direct-labeled specific probe was used, resulting in a blue signal.

The DNA denaturation of sperm and probes, incubation, posthybridization washing, and nuclear staining were performed according to the Kreatech protocol. After preparation, slides were observed using a fluorescence microscope (Nikon Eclipse E600) equipped with a triple band-pass filter set [fluorescein isothiocyanate (FITC), tetrarhodamine isothiocyanate (TRITC), 6-diamino-2-phenylindole (DAPI)]. Single spots were evaluated as reported elsewhere (Robbins et al., 1995). For each patient, at least 2,500 cells were scored.

### Acridine Orange Test (AO Test)

The AO test was used to assess the heterogeneity of sperm DNA. Briefly 10 μl of semen sample and equal volume of AO solution [10 mL acridine orange stain (1%) was mixed with 2.5 mL Na2HPO4.7H2O (0.3 mol/l), and 40 mL citric acid (0.1 mol/l)] were mixed together on the surface of a glass slide and covered with a glass coverslip. The sample was then evaluated with a fluorescence microscope with a 490 nm excitation light and 530 nm barrier filter. Nuclei from 200 spermatozoa were examined and scored as with green- or red-fluorescence. When the head showed green fluorescence, sperm were considered normal (double-stranded DNA); they were considered denatured when fluorescence was red (single-stranded DNA). Results were expressed as the percentage of sperm that showed altered (red) fluorescence (Eggert-Kruse et al., 1996).

### TUNEL Assay

The DNA fragmentation was evaluated by TUNEL assay performed with the Cell Death Detection Kit (Roche Diagnostics). Fluorescein isothiocyanate–deoxyuridine triphosphate was used as label according to the manufacturer’s instructions and counterstained with 40,6-diamidino-2-phenylindole. Briefly, after preparation, sperm samples were smeared on microscope slides, fixed with 4% paraformaldehyde, and permeabilized with 0.1% Triton X-100 in 0.1% sodium citrate. The specimens were incubated in TUNEL reaction mixture in a humidified atmosphere for 60 min at 37°C in the dark. The slides were rinsed twice, and then 1 mL 40,6-diamidino-2-phenylindole was added to counterstain the cells. Four hundred spermatozoa were analyzed at 40 by using a Nikon E600 epifluorescence light microscope equipped with a 450–490-nm excitation (Nikon), and those cells with green fluorescence were considered to be altered.

### γH2AX Flow-Cytometric Analysis

The sperm cells were fixed, permeabilized, washed in PBS and centrifuged as reported in the previous paragraph. Thereafter, pellets were resuspended in 200 μl of PBS-0.1% BSA and equally split in two vials used as samples and negative controls. The staining for γH2AX was conducted resuspending the pellet samples in 200 μl of polyclonal rabbit anti-γH2AX antibody (1:200 dilution), (Millipore, Billerica, MA, USA) for 90 min at 37°C. Negative controls and samples were then rinsed in PBS-0.1% BSA solution and after centrifugation were resuspended in 200 μl of secondary goat anti-rabbit IgG-fluorescein conjugated antibody (1:400 dilutions; Millipore, Billerica, MA, USA) for 45 min at 37°C. Finally, the sperm cells were rinsed once in PBS-0.01% tween-20 solution (Sigma–Aldrich, St. Louise, MO, USA) for 5 min at room temperature and centrifuged. Pellet was washed once in a PBS solution for 5 min at room temperature and centrifuged. Before analysis with a Flow Cytometer cells were resuspended in 300 μl of Propidie Iodide solution (25 ng/m of PI in H_2_O; Sigma–Aldrich, St. Louise, MO, USA). Samples were then analyzed on a FACScan flow cytometer (Becton Dickinson, Mountain View, CA, USA) equipped with a 15 mW – 488 nm, air cooled, argon ion laser for excitation. For each sample, 40 000 events were recorded within the characteristic flame-shaped region in the forward scatter/side scatter (FSC/SSC) dot plot. Data were acquired and analyzed with Cellquest analysis software. The average amount of γH2AX antibody staining relative to the untreated control was scored as the percentage of spermatozoa having fluorescence intensities above a threshold excluding ≤1% of the events in the negative sample (Li et al., 2006). Results were expressed as percentage of DSBs in total sperm analyzed.

### Statistical Analysis

Results are expressed as mean ± SD. Variations in semen and hormonal parameters and testicular volume were considered and are expressed as raw differences.

Comparisons among four groups were performed through ANOVA test. To evaluate effects of therapy along time, two-sample *t*-tests were computed using T0 as reference time point. To compare therapies, also considering the initial individual characteristics, a repeated measures ANOVA is performed for each parameter of interest. The software package R (R Core Team, 2012) was used for statistical analyses. Probability values of <0.05 were considered statistically significant.

## Results

Mean age of different groups was not significantly different (*S* = 32.0 ± 1.4, CARB = 34.2 ±_3.5, BEP = 34.0 ± 2.8 years).

In **Table 1** are reported seminal parameters, hormonal levels, and testicular volume at different time points of the study of surveilled patients (*S*) and patients treated with CARB or BEP observed at different time points of the study. At baseline, seminal parameters, hormonal status, and testicular volume did not differ between three groups. At T1 the BEP group had a significant reduction of sperm concentration and sperm count compared with baseline (*p* < 0.0001) and versus both CARB and *S*-group (*p* < 0.0001). At T2, these semen alterations were still evident in BEP patients compared with other groups. All groups showed no significant variations in sperm motility and morphology during the whole study. After orchiectomy, all groups showed a progressive increase of FSH plasma levels. In treated patients (both CARB and BEP groups), this change was already significant at T1 (*p* < 0.001 versus T0) while *S*-group showed a significant increase only at T2. In contrast, LH, total testosterone, and testicular volume remained unchanged in all groups.

**Table 1:** Semen parameters, hormones, and testicular volume observed in different groups at baseline, T1 and T2.

	Seminal parameters				Hormonal parameters			Testicular volume (cc)
			
	Sperm concentration (million/mL)	Sperm count (million)	Progressive motility (%)	Normal morphology (%)	FSH (mU/ml)	LH (mU/ml)	Total T (nmol/L)	
S-T0	47.78 ± 52.72	132.6 ± 127	36.4 ± 20.9	14.2 ± 6.1	8.1 ± 4.56	5.52 ± 2.7	16.6 ± 5.2	17.2 ± 5.2
S-T1	61.88 ± 50.63	165.5 ± 117	50.48 ± 18.9	12.52 ± 7.3	8.6 ± 3.7	5.7 ± 2.9	16.9 ± 4.6	19.9 ± 3.4
S-T2	68.9 ± 49.85	179.1 ± 136.9	47.9 ± 16.0	12.78 ± 7.0	13.68 ± 7.9^∗^	6.8 ± 2.2	17.5 ± 5.5	20.5 ± 4.3
Carb-T0	48.4 ± 48.4	134.6 ± 150	37.8 ± 16.1	13.2 ± 10.7	8.8 ± 5.3	5.7 ± 2.6	16.1 ± 5.7	16.6 ± 4.1
Carb-T1	49.14 ± 45.2	139.0 ± 140.2	44.8 ± 20.9	11.38 ± 4.2	12.0 ± 6.5^∗^	5.8 ± 2.7	14.9 ± 3.9	17.9 ± 3.9
Carb-T2	60.22 ± 54.43	159.6 ± 124.1	43.9 ± 18.2	13.9 ± 4.8	12.1 ± 6.0^∗^	5.9 ± 2.4	14.6 ± 3.8	18.2 ± 3.8
BEP-T0	46.02 ± 62.4	128.2 ± 149.6	35.4 ± 21.1	14.5 ± 7.2	8.1 ± 5.2	5.8 ± 2.6	17.5 ± 6.3	16.0 ± 4.9
BEP-T1	15.17 ± 21.9^∗^#	32.55 ± 46.0^∗^#	26.5 ± 15.5	11.4 ± 7.0	14.5 ± 9.6^∗^	8.1 ± 4.2	13.7 ± 5.1	14.12 ± 4.2
BEP-T2	25.44 ± 27.7°	66.03 ± 74.5°	37.9 ± 17.2	15.6 ± 9.3	13.9 ± 8.6^∗^	7.1 ± 3.5	16.8 ± 4.9	15.94 ± 4.8


*S, surveillance; Carb, carboplatin; BEP, bleomicin, etoposide, and platinum; T0, after orchiectomy; T1, 1 year after orchiectomy or after end of CT; T2, 2 years after orchiectomy or after end of CT. ^∗^*p* < 0.0001 vs. T0, #*p* < 0.0001 vs. Carb T1 and S-T1, °*p* < 0.0001 vs. Carb T2 and S-T2.*

**Table 2** shows results of FISH test to evaluate sperm aneuploidies (autosomes, sex chromosomes, and total aneuploidies), acridine orange, TUNEL and γH2AX tests to evaluate, respectively, DNA compaction, single- or double-strand fragmentation and double-strand fragmentation. No differences between groups were observed at baseline. When, we considered FISH analysis for autosome and sex chromosomes separately data were not different in all groups along the study. However, considering autosomes and sex chromosomes as a whole, BEP subjects showed significantly higher sperm aneuploidies at T1 (*p* < 0.01 vs. T0 and CARB- group). This alteration was slightly reduced at T2, even if the aneuploidy rate in BEP group was still significantly higher than in other groups (*p* < 0.05). Similarly to sperm aneuploidies, at T1 sperm DNA compaction and fragmentation (both single- and double strand breaks) of BEP group were significantly impaired compared to baseline, S- and CARB group. These alterations were persistent after 2 years from the end of BEP treatment. No impairments of sperm aneuploidies, DNA compaction and fragmentation were observed in the CARB group both after 1 and 2 years from the end of treatment. In this study, we found no factor able to predict changes of sperm parameters, sperm nucleus, and hormones in the response to CT.

**Table 2:** Sperm aneuploidies and nuclear status observed in different groups at baseline, T1 and T2.

	FISH (Aut.) (%)	FISH (Sex) (%)	FISH (Tot.) (%)	Acridine orange (%)	TUNEL (%)	γH2AX (%)
S-T0	0.61 ± 0.32	0.73 ± 0.52	1.34 ± 0.76	19.78 ± 4.0	16.13 ± 4.16	5.35 ± 3.35
S-T1	0.64 ± 0.27	0.68 ± 0.28	1.33 ± 0.47	19.52 ± 3.59	15.74 ± 4.36	6.04 ± 3.3
S-T2	0.65 ± 0.29	0.65 ± 0.30	1.29 ± 0.44	18.87 ± 4.05	15.09 ± 4.66	6.13 ± 3.14
Carb-T0	0.69 ± 0.26	0.72 ± 0.36	1.41 ± 0.50	20.21 ± 4.4	16.38 ± 3.88	6.12 ± 2.88
Carb-T1	0.69 ± 0.19	0.61 ± 0.24	1.30 ± 0.35	18.83 ± 4.65	16.21 ± 4.1	6.79 ± 3.93
Carb-T2	0.69 ± 0.22	0.60 ± 0.27	1.29 ± 0.36	17.29 ± 4.68	16.0 ± 4.53	6.58 ± 3.75
BEP-T0	0.69 ± 0.35	0.8 ± 0.4	1.49 ± 0.68	20.18 ± 5.64	16.94 ± 5.22	6.97 ± 4.58
BEP-T1	1.08 ± 0.61	1.19 ± 0.78	2.27 ± 1.38^∗#^	25.43 ± 6.17^∗#^	21.49 ± 4.94^∗#^	11.42 ± 4.28^∗#^
BEP-T2	0.83 ± 0.41	0.88 ± 0.54	1.9 ± 0.89^∘$^	22.42 ± 5.02^∗$^	20.64 ± 5.2^∗#^	10.09 ± 3.34^∗∗^


*Aut, autosomes (13,18,21); Sex, sex chromosomes (X,Y); Tot., all chromosomes; *S*, surveillance; Carb, carboplatin; BEP, bleomicin, etoposide, and platinum; T0, after orchiectomy; T1, 1 year after orchiectomy or after end of CT; T2, 2 years after orchiectomy or after end of CT. ^∗^*p* < 0.0005 vs. T0, ^#^*p* < 0.0005 vs. Carb T1, °*p* < 0.01 vs. T0, ^$^*p* < 0.01 vs. carb T2, ^∗∗^*p* < 0.05 vs. T0 and Carb T2.*

**FIGURE 1:**
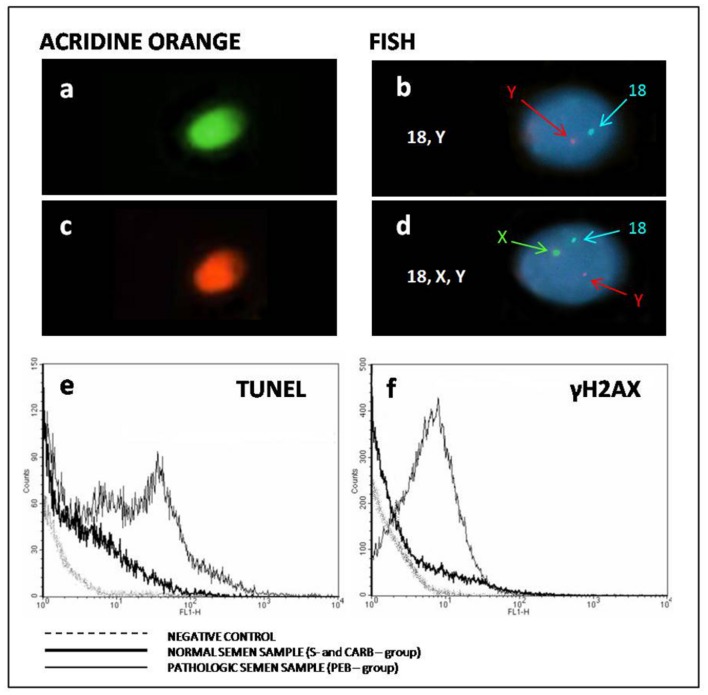
**Example of different tests performed to evaluate sperm nuclear status in surveillance and chemotherapy (CT) treated patients (Carb and PEB).** **(a,c)** Examples of normal and altered sperm compaction. **(b,d)** Examples of euploid (18, Y) and aneuploid sperm (18, X, and Y). **(e)** Examples of single/double strand breaks observed in different groups. **(f)** Examples of double strand breaks observed in different groups.

In **Figure 1** are reported some examples of different tests performed to evaluate sperm nuclear status in surveillance and CT treated patients. **Figures 1c,d** and thin lines of **Figures 1e,f** show examples of sperm from BEP patients.

## Discussion

Testicular cancer is the most frequent malignancy affecting males at reproductive age. It has been reported that a significant number of European subjects over reproductive age develop a TGCTs every year (Znaor et al., 2014) and its incidence is increasing in several countries over the past 50 years (Huyghe et al., 2007). Actually the new therapeutic strategies and follow-up allow survivorship in over 95% of cases. After inguinal orchiectomy patients can be treated by RT on pelvic lymph nodes, CT or simply surveilled on the basis of tumor histology, staging, prognostic factors, and patients decision after oncological counseling. Combined therapy with etoposide, cisplatin, and bleomycin is largely considered a useful and effective option for the treatment of TGCTs (Oldenburg et al., 2013).

In 2005 it has been introduced the possibility of treatment with CARB alone in selected cases of TGCTs (seminomas and first stage of disease). Moreover, it has been demonstrated the non-inferiority of such treatment compared with RT, especially with regard to the disease-free period (Oliver et al., 2005). The risk of second malignancy like non-testicular germ cell has been associated with moderate-dose infradiaphragmatic RT in stage I seminoma. Further data by a subsequent study have later confirmed these findings (Oliver et al., 2011) and were followed by a broad scientific debate on the effectiveness of one cycle of CARB compared with RT. In particular, the matter of debate was the methodology to study renal function for decisions about CARB dose (Dieckmann et al., 2011; Stenning et al., 2011). Recently, this aspect has been definitely clarified and the gold standard for GFR measurement and consequently dose calculation of adjuvant CARB CT is considered (51)Cr-EDTA (Quinton et al., 2013). Based on this evidence, the number of people suffering from TGCTs treated with one cycle CARB alone has increased in recent years.

It is far known that conventional CT regimens used in TGCTs patients as BEP, may affect male fertility and endocrine function of the residual testis (Hassold et al., 1996; Tempest et al., 2008; Bujan et al., 2013). Moreover, BEP use has been reported to affect sperm chromosomes and to alter DNA status (De Mas et al., 2001; Zhou and Bartek, 2004; Liu et al., 2015; Paoli et al., 2015). In contrast, no data are available regarding the impact of treatment with CARB on gonadal function. In this preliminary study, we evaluated for the first time the effect of one cycle CARB on semen parameters, sex hormones, sperm aneuploidies, and DNA status in a group of TGCTs patients treated with BEP or one cycle of CARB followed at 12 (T1) and 24 months (T2) after therapy. A third group of TGCTs patients in which oncologist suggested only surveillance after orchiectomy, served as a control group (*S*-group). Our results confirmed that BEP treatment has a detrimental effect on sperm parameters. In contrast, therapy with CARB alone seems to not affect semen quality. In fact, as in surveillance patients, semen parameters, hormones, and volume of the residual testis remained unchanged. Moreover, our findings showed an increase of sperm aneuploidies after BEP-therapy and these alterations persisted upon 24 months after the end of treatment. Moreover, we observed negative effects of BEP therapy on sperm DNA integrity, persisting upon 24 months from the end of treatments. In our experience, CARB use did not affect sperm aneuploidies, DNA compaction, and DNA fragmentation at any time of the study. Basing also on these results and the risk of second tumors (non-TGCT0), in patients treated with RT, the use of CARB mono-CT is suggested in selected patients (tumor histology, stage of disease, and prognostic factors) with TGCTs.

## Conclusion

Our results although preliminary, denote that CARB alone seems not to have a negative effect on fertility and sex hormones in selected TGCTs patients. Despite the small amount of patients and the need of larger studies to confirm our data, the present study is the first on this topic suggesting that one cycle CARB, besides representing a valid therapeutic option, seem to not affect spermatogenesis, sperm aneuploidies, DNA integrity, and gonadal hormones in TGCTs orchidectomized patients. Moreover, the multidisciplinary approach is mandatory to choose the best treatment in patients with cancer disease potentially curable (Berretta et al., 2014).

## Author Contributions

Selected the patients: MG, MB, AG, PP, and RS. Conceived and designed the experiments: MG, AG, and CF. Performed the experiments: AB, PP, BS, and IC. Analyzed the data: MG, LF, and AG. Contributed reagents/materials/analysis tools: AB, IC, BS, and PP. Wrote and revised the manuscript: MG, MB, CF, and AG.

## Conflict of Interest Statement

The authors declare that the research was conducted in the absence of any commercial or financial relationships that could be construed as a potential conflict of interest.
